# The role of human 5-Lipoxygenase (5-LO) in carcinogenesis - a question of canonical and non-canonical functions

**DOI:** 10.1038/s41388-024-03016-1

**Published:** 2024-04-04

**Authors:** Astrid S. Kahnt, Ann-Kathrin Häfner, Dieter Steinhilber

**Affiliations:** Institute of Pharmaceutical Chemistry, Goethe University, Max-von-Laue-Straße 9, 60438 Frankfurt/Main, Germany

**Keywords:** Inflammation, Non-coding RNAs, Drug development

## Abstract

5-Lipoxygenase (5-LO), a fatty acid oxygenase, is the central enzyme in leukotriene (LT) biosynthesis, potent arachidonic acid-derived lipid mediators released by innate immune cells, that control inflammatory and allergic responses. In addition, through interaction with 12- and 15-lipoxgenases, the enzyme is involved in the formation of omega-3 fatty acid-based oxylipins, which are thought to be involved in the resolution of inflammation. The expression of 5-LO is frequently deregulated in solid and liquid tumors, and there is strong evidence that the enzyme plays an important role in carcinogenesis. However, global inhibition of LT formation and signaling has not yet shown the desired success in clinical trials. Curiously, the release of 5-LO-derived lipid mediators from tumor cells is often low, and the exact mechanism by which 5-LO influences tumor cell function is poorly understood. Recent data now show that in addition to releasing oxylipins, 5-LO can also influence gene expression in a lipid mediator-independent manner. These non-canonical functions, including modulation of miRNA processing and transcription factor shuttling, most likely influence cancer cell function and the tumor microenvironment and might explain the low clinical efficacy of pharmacological strategies that previously only targeted oxylipin formation and signaling by 5-LO. This review summarizes the canonical and non-canonical functions of 5-LO with a particular focus on tumorigenesis, highlights unresolved issues, and suggests future research directions.

## Introduction

5-lipoxygenase (5-LO) is the key enzyme in the formation of leukotrienes (LT), potent pro-inflammatory lipid mediators, as well as some of the oxylipins termed specialized pro-resolving lipid mediators (SPMs) that are suggested to contribute to the resolution of inflammation. Interestingly, the enzyme is not only present in the cytosol of a cell, but is also able to translocate to nuclear compartments where it is associated with the euchromatin. In addition to its well characterized catalytic activities involved in the formation of various lipid mediators, more recent data suggest that the 5-LO protein itself has additional cellular functions beyond its oxygenase activity which contribute to the immunological role of the enzyme. These non-canonical activities include the transcriptional regulation of genes as well as the regulation of the generation of distinct miRNAs by Dicer. They appear to play an important role in the development and maintenance of human cancers, where they are closely intertwined with the catalytic functions of the enzyme. This review aims to provide an overview of the role of 5-LO in carcinogenesis and to shed light on the possible influence of canonical and non-canonical functions.

## Functions of 5-LO

### Oxylipin formation (canonical function)

**Fig. 1:**
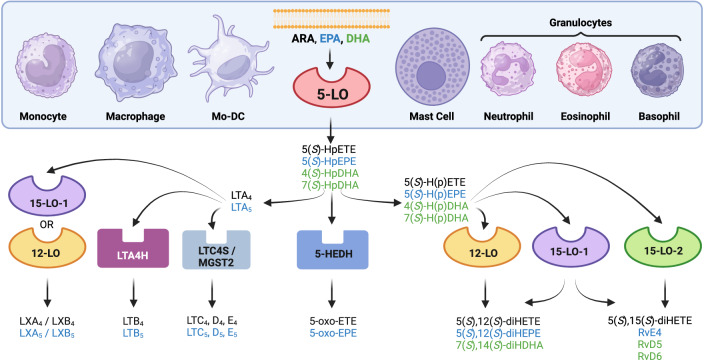
5-LO-dependent oxylipin formation from ARA, EPA and DHA. In healthy individuals, 5-LO is mainly expressed in leukocytes (light blue box). The preferred substrate of 5-LO is ARA (in black). The enzyme also accepts a number of other lipids as substrates, including EPA (in blue) and DHA (in green) as well as certain monohydroxylated oxylipins such as HETEs (pathway not depicted here). The enzymes downstream of 5-LO are expressed either in the same cell or in a second cell type (transcellular biosynthesis). ARA arachidonic acid, DHA docosahexaenoic acid, EPA eicosapentaenoic acid, HDHA hydroxydocosahexaenoic acid, 5-HEDH 5-hydroxyeicosanoid dehydrogenase, HEPE hydroxyeicosapentaenoic acid, HETE hydroxyeicosatetraenoic acid, HpDHA hydroperoxydocosahexaenoic acid, HpETE hydroperoxydocosahexaenoic acid, HpEPE hydroperoxyeicosapentaenoic acid, LT leukotriene, LTA4H LTA_4_ hydrolase, LTC4S LTC_4_ synthase, LX lipoxin, LO lipoxygenase, MGST2 microsomal glutathione S-transferase 2, Mo-DC monocyte-derived dendritic cell, Rv Resolvin.

The 5-LO pathway was discovered in the seventies of the last century where it was found that arachidonic acid (ARA) can be oxidized at carbon C(5) which finally leads to the generation of 5(*S*)-HETE, 5-oxo-ETE and the LTs [1, 2]. Later, the enzymes actually involved in the pathway among them 5-LO, its down-stream LT synthases LTA_4_ hydrolase and LTC_4_ synthase as well as other enzymes involved in the generation of leukotriene B_4_ (LTB_4_) and the cysteinyl-containing leukotrienes LTC_4_, D_4_ and E_4_ were characterized [3]. Investigation of the biological functions of these lipid mediators revealed that LTs are mediators of inflammatory and allergic responses, are part of the innate immune system and are involved in host defense reactions [3–8]. Although ARA is by far the preferred substrate, 5-LO can also metabolize certain oxidized ARA derivatives (e.g. 12- and 15-HETE) as well as the omega-3 fatty acids eicosapentaenoic acid (EPA) and docosahexaenoic acid (DHA) (Fig. 1). In particular, the formation of DHA-derived oxylipins is often low. 5-LO was also reported to be involved in the generation of specialized pro-resolving lipid mediators (SPMs) such as lipoxins and resolvins [9]. Figure 1 shows the biosynthesis pathway of 5-LO-dependent oxylipins. However, whereas formation of LTs, 5-HETE and 5-oxo-ETE and their signaling via the respective receptors (BLT1, CysLT1, CysLT2, OXER1) by cells and tissues has been clearly demonstrated, the endogenous biological role of many of the SPMs has recently been challenged: Most of the 5-LO-derived lipoxins and resolvins are hardly produced by leukocytes and only dihydroxylated SPMs such as RvE4 and RvD5 have been consistently detected [10], the proposed receptors are not validated up to now [11], and many publications reporting SPM analyses in biological samples used flawed procedures for peak identification and quantification by LC-MS/MS [12].

The cellular activity of 5-LO is tightly regulated in the cell and its expression is mainly restricted to immune cells such as granulocytes, monocytes/macrophages, mast cells, dendritic cells and B lymphocytes whereas platelets, endothelial cells and erythrocytes are 5-LO negative [13]. In the skin, Langerhans cells strongly express 5-LO [14].

In leukocytes, 5-LO is a long-lived protein which is activated by calcium and phosphorylation [15]. In the absence of exogenously added substrate, the formation of 5-LO metabolites in leukocytes is associated with the translocation of 5-LO from the cytosol or from soluble compartments of the nucleus to the nuclear membrane. Here, the enzyme interacts with the 5-LO activating protein (FLAP), which is required for the conversion of 5-LO metabolites from ARA, EPA, 15(*R/S*)-HETE and 17(*R/S*)-HDHA [16–19] whereas oxygenation of DHA can occur independently of FLAP [20].

The cellular localization of 5-LO (nuclear or cytosolic) is stimulus- and cell type-dependent and is controlled by at least three nuclear localization sequences and one nuclear export sequence [21, 22], which in part contain phosphorylation sites so that their function is regulated by phosphorylation. These post-transcriptional modifications have divergent consequences on the subcellular localization and activity of 5-LO. In vitro, protein kinase (PK)A was found to phosphorylate 5-LO at Ser523 [23]. p38 mitogen-activated protein kinase-activated protein kinase (MAPKAPK, MK)-2/-3, PKA, and CaMK-II phosphorylate the enzyme at Ser271. ERK-1/-2 trigger phosphorylation at Ser663 [24, 25]. Phosphorylation at Ser523 inhibits the activity of 5-LO and leads to its cytosolic localization, obviously by inhibiting the function of the nuclear localization sequence located at Ser523 [26]. Phosphorylation at Ser271 suppresses nuclear export of 5-LO by inhibition of the nuclear export sequence at Ser271, so that the nuclear localization of 5-LO is maintained [22]. The signal that leads to nuclear import of 5-LO is unknown up to now.

In addition to phosphorylation of serine residues, the tyrosine kinases Fgr, HCK and Yes were found to phosphorylate Tyr42, Tyr53 and either Tyr94 or Tyr445 of 5-LO in vitro [27]. However, it is unclear whether these phosphorylations occur in vivo, their physiological consequences are not yet known.

### Non-canonical functions

In resting alveolar macrophages, nuclear 5-LO is associated with euchromatin [28]. Since this function is irrelevant for LT formation, it was not known for a long time why the enzyme is found in transcriptionally active regions of the human genome. Furthermore, 5-LO is known to interact with other proteins such as Dicer and coactosin-like protein (CLP) [29]. While CLP influences the catalytic activity of 5-LO by acting as a chaperone for the enzyme thereby upregulating the production of LTA_4_ [30, 31], the interaction of 5-LO with Dicer has been enigmatic. In recent years, however, several publications have shown that the 5-LO protein, independent of its function as a fatty acid oxygenase, is involved in regulation of gene expression either directly or through interaction with other proteins.

#### Influence on microRNA expression and processing

**Fig. 2:**
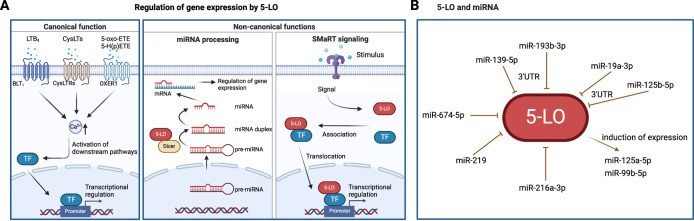
Influence of 5-LO on gene expression. **A** Both, 5-LO-derived lipid mediators (canonical pathway) as well as non-canonical functions of the enzyme can influence the gene expression in cancer cells. In the canonical pathway, oxylipins mediate their effects via receptor interaction and the subsequent activation of signaling pathways that lead to the regulation of transcription. The non-canonical functions include on the one hand, the influence of 5-LO on miRNA processing by Dicer which then leads to an altered cellular miRNA profile and consequently to a regulation of gene expression. On the other hand, 5-LO can act as STRaND (**s**huttling **t**ranscriptional **r**egulator **a**nd **n**on-**D**NA binding) in the SMaRT (**S**ensing external signal, acting as **m**essenger **a**nd **r**egulating **t**ranscription) signaling pathway. Here, the enzyme interacts with transcription factors and shuttles them into the nucleus to regulate transcription. **B** Regulation of 5-LO expression by miRNAs and vice versa.

Years ago, 5-LO was found to form a protein-protein interaction with the C-terminal domain of Dicer, the central endoribonuclease in the small RNA pathway, which is responsible for the conversion of pre-miRNAs into miRNA duplexes in the cytosol (Fig. 2) [32]. miRNAs are highly conserved small non-coding ribonucleic acids that regulate gene expression at the post-transcriptional level. Binding of miRNAs to their target mRNAs can lead to translational repression or mRNA degradation. Furthermore, they can act as decoy for RNA-binding proteins [33, 34] or even as ligands for the toll-like receptor family [35, 36]. This interaction of 5-LO with Dicer was later demonstrated in situ in the monocytic cell line MonoMac-6 [37]. Here, using 5-LO knockout cells, two remarkable effects of 5-LO on miRNA biogenesis were found in differentiated MonoMac-6 cells. First, the presence of 5-LO enhances the expression of the pri-miR cluster miR-99b/let-7e/miR-125a, and second, the 5-LO protein has a direct effect on the function of Dicer by downregulating the processing of pre-let-7e, leading to an increase in miR-125a and miR-99b but not in let-7e. In addition, the 5-LO knockout was found to affect other miRNAs such as let-7a and let-7b-5p, members of the let-7 family, which are highly conserved between species and play an important role in tumorigenesis and developmental processes.

Vice versa, 5-LO and other proteins in the ARA cascade are also targets of miRNAs and can be regulated by them. miR-674-5p seems to have a negative regulating effect on 5-LO expression in concanavalin A-induced autoimmune reactions in mouse liver [38] whereas miR-219 down-regulates 5-LO in macrophages [39]. Furthermore, miR-19a-3p and miR-125b-5p bind to the 3’UTR of 5-LO mRNA and regulate the expression of 5-LO protein in different immune cells in a stimulus-specific manner [40, 41] whereas binding of miR-193b-3p and miR-139-5p to the 3’UTR of 5-LO mRNA was demonstrated in a cerebral focal I/R injury rat model [42] and in chronic unpredicted mild stress-induced depressed rats, respectively [43]. In addition, miR-216a-3p was shown to inhibit colorectal cancer proliferation by targeting 5-LO and cyclooxygenase-2 (COX-2) [44].

It is interesting to note that not only 5-LO is regulated by miRNAs, but also FLAP-, which transfers the substrate ARA to 5-LO and which is necessary to obtain 5-LO products in intact cells [45]. Besides targeting COX-2 in lung cancer cells, miR-146a is also a negative regulator of FLAP in lung cancer resulting in a dual inhibition of the ARA metabolism in these cells [46]. In HepG2 liver cells, miR-146a-mediated down-regulation of FLAP mRNA has a stimulatory effect, leading to increased migration [45]. Furthermore, miR-135a and miR-199a-5p bind the 3’UTR of FLAP mRNA resulting in a negative regulation in pulmonary microvascular endothelial cells [47]. In addition, decreased levels of miR-335 and miR-495 targeting the 3’UTR of FLAP were found to lead to upregulation of FLAP during ischemic stroke [48]. Figure 2B depicts the miRNAs involved in 5-LO expression as well as the miRNAs influenced by 5-LO.

#### 5-LO as STRaND (shuttling transcriptional regulator and non-DNA binding)

For some time now, there has been increasing evidence that 5-LO, in addition to its known function as a metabolizing enzyme in the ARA pathway, can influence the gene expression of various target genes not only via its metabolites but also directly. As mentioned above, 5-LO is able to translocate from the cytosol to the nucleus. Therefore, 5-LO has the potential to function as STRaND (Fig. 2A) [49]. STRaNDs bind to transcription factors in the cytosol, migrate with them into the nucleus and bind indirectly to the DNA.

First hints to this were found when 5-LO was detected in Rel-A (p65) immunoprecipitate fractions of lysates of the human leukemic cell line HL-60 [50]. Rel-A is part of the NFκB family, transcription factors which are well-known regulators of the immune system addressing inflammatory pathways. The NFκB family consists of five proteins and can be divided into two classes: Class 1 consists of p105/p50 and p100/p52, which are produced as larger precursors and then cleaved to form the smaller subunits. They contain ankyrin repeats at the C-terminus, which are responsible for transrepression. Class 2 consists of the members Rel-A, Rel-B and c-Rel, which contain a transcriptional activation domain region at the C-terminus. All members of the NFκB family have a Rel homology region at their N-terminus, which comprises around 300 amino acids. This region is responsible for important functions such as dimerization, interaction with IκB, DNA binding and cellular localization [51]. In order to be transcriptionally active, two different NFκB subunits dimerize, translocate into the nucleus and bind to response elements on the DNA.

In addition to interaction with Rel-A, several other protein-protein interactions have been found for various members of the NFκB family with 5-LO. Data published in human interactome databases show interaction of 5-LO and c-Rel as well as 5-LO and p100/p52 [52–54]. Recently, we found that the 5-LO protein is a regulator of NFκB target genes in the monocytic cell line MonoMac-6 [55]. After differentiation with TGFβ/1,25(OH)_2_D_3_ and subsequent LPS stimulation of the cells we could show that the 5-LO knock-out led to a complete loss of kynureninase protein expression, which is encoded by the NFκB target gene *KYNU*. The subsequent 5-LO knockin of either 5-LO wild type protein or a catalytically inactive 5-LO mutant resulted in the restoration of kynureninase mRNA and protein expression. COX-2 which is encoded by the NFκB-controlled *PTGS2* gene, representing an important enzyme in the ARA metabolism pathway, showed the opposite effect: The knockout of 5-LO resulted in an upregulation of COX-2 mRNA and protein whereas re-expression of 5-LO led to a downregulation. Furthermore, a combined ChIP-Seq/FAIRE-Seq analysis revealed an association of 5-LO to euchromatin in close proximity to transcription start sites in MonoMac-6 cells, suggesting that the enzyme might stabilize euchromatin or acts as chromatin remodeler. Moreover, an enrichment analysis of publicly available ChIP-Seq datasets revealed that 5-LO co-localizes with histone marks such as H3K27 acetylation or H3K4 methylations, which are typical for activating and enhancing DNA regions. Among the transcription factors, the RNA binding protein RBFOX2 and the bromodomain-containing proteins BRD2 and 4 showed the greatest overlap with the 5-LO peaks. Interestingly, BRD4 is a known co-activator of NFκB that binds to acetylated p65, thus providing an additional link of 5-LO/p65 regulation [56].

In addition to its interaction with euchromatin and NFκB family members, a direct interaction of 5-LO and RUNX2, a transcription factor that plays an important role in differentiation, growth processes and cell cycle regulation, was recently detected [57]. In patients suffering from cardiac hypertrophy and heart failure, 5-LO protein levels are significantly increased while levels of the 5-LO product 5-HETE were hardly changed. RNA-Seq of cardiomyocytes derived from adult wild type and 5-LO knockout mice showed a strong correlation between 5-LO and MAPK/EGFR signaling pathways. Immunoprecipitation of Flag-tagged 5-LO in neonatal rat ventricular cardiomyocytes confirmed a direct binding of 5-LO to the transcription factor RUNX2. 5-LO silencing revealed that 5-LO promotes the nuclear transport of RUNX2 independent of its enzymatic activity. In addition, 5-LO increased the expression of EGFR by RUNX2 in cardiomyocytes causing cardiac remodeling. Interestingly, RUNX2 interacts with and is regulated by BRD4 [58, 59], suggesting the involvement of 5-LO in a transcriptional complex of multiple proteins with BRD4, RUNX2 and 5-LO. Figure 2 summarizes the non-canonical functions of 5-LO.

## 5-LO and cancer

### Hemato-oncological diseases

It has been shown that 5-LO expression is deregulated in different types of leukemia. Peripheral CD34^+^ acute myeloid leukemia (AML) cell clones express 5-LO while healthy CD34^+^ cells do not [60, 61]. Interestingly, 5-LO was not active in some of the clones. Only AML clones showing a certain degree of differentiation (monoblasts, pro-myelocytes, monocytes) showed substantial LTB_4_ formation [60]. Mantle cell lymphomas express higher levels of 5-LO compared to mantle zone B cells from healthy individuals. Of note, 5-LO activity was quite low and dependent on the presence of diamide in leukemic B cells as well as in B cells from healthy donors [62]. In contrast to lymph node mantle zone-derived B cells, 5-LO expression in cells located within the germinal center of the lymph node is usually low in healthy individuals. In contrast, follicular lymphomas derived from germinal center B cells overexpress 5-LO [63].

Hemato-oncological malignancies often carry chromosomal abnormalities such as deletions or translocations leading to oncogenic fusion proteins or oncogene activation by acquisition of new promoter or enhancer sites. 5-LO expression is potently triggered by the gene products generated by the most frequent translocation events in leukemic cells. This has been shown for RUNX1-ETO9a, the result of a common chromosomal abnormality in AML, which upregulates 5-LO expression via KLF6. Interestingly, loss of 5-LO expression reduces activity of RUNX1-ETO9a, MLL-AF9 and PML-RARα in vitro suggesting a feedback loop [64]. We could also show that the fusion protein AF4-MLL, involved in high risk lymphoblastic leukemias, enhances *ALOX5* transcript elongation more potently compared to AF4 and MLL wildtype proteins [65]. Furthermore, MLL-AF4 potently stimulates 5-LO promoter activity [66].

It has been shown that expression of 5-LO gives a survival advantage to leukemic cells. 5-LO and LTA4H protein expression correlates with the clinical evolution of chronic B cell lymphomas (B-CLL), being highest in patients with rapidly progressing disease. In line with this, proliferation and survival of aggressive B-CLL cells was dependent on the LTB_4_/BLT2 axis [67, 68]. Moreover, white blood cell preparations of CML (chronic myeloid leukemia) patients display an increased LTC_4_ biosynthesis capacity which is not due to elevated numbers of classical CysLT-producing leukocytes such as monocytes and eosinophils [69]. Further investigations could show that this elevated CysLT capacity of CML cells was due to leukemic CD16^+^ neutrophils overexpressing LTC4S. Here, LTB_4_ production was not elevated compared to healthy controls [70]. Furthermore, Imatinib treatment has been shown to normalize the aberrant LTC_4_ production in CD16^+^BCR-ABL^+^ cells from CML patients [71].

In addition to peripheral leukemic cells, a number of reports could show that 5-LO is a critical regulator of leukemic stem cells. 5-LO products dose-dependently stimulate myeloid stem cell proliferation [72] and a mouse model of BCR-ABL-induced CML could show that absence of 5-LO prevents CML induction by BCR-ABL due to a defect in leukemic stem cell differentiation, division and long-term survival. Treatment with the 5-LO inhibitor Zileuton had the same effect [73]. In keeping with this, we could show that pharmacological inhibition of 5-LO also affects the aberrant stem cell capacity in a PML/RARα-positive model of AML (acute myeloid leukemia) and a murine cancer stem cell (CSC) model [Sca-1(+)/lin(−) murine hematopoietic stem and progenitor cells retrovirally transduced with PML/RARα]. This was due to the inhibition of the PML/RARα-mediated activation of Wnt signaling in the stem cells. Interestingly, the knock-out of 5-LO did not recapitulate the inhibitor data. When we investigated this further, we could show that inhibition of Wnt signaling is mediated by an enzymatically inactive form of 5-LO, which binds to β-catenin thereby hindering nuclear translocation and activation of target genes clearly pointing to a role of 5-LOs non-canonical functions in leukemogenesis [74].

In contrast to these studies, two other groups found only low levels of 5-LO in bone marrow-derived BCR^-^ABL-CD34^+^CD38^-^ cells from CML patients [75, 76]. Furthermore, neither Zileuton nor Montelukast had an impact on cell growth of bone marrow-derived BCR-ABL-CD34^+^CD38^-^ cells from CML patients in long-term culture-initiating cell and colony assays [75]. This lack of efficacy of 5-LO inhibitors in the maintenance of leukemic stem cells clearly indicates that this effect is not dependent on 5-LO’s catalytic activity but rather points to non-canonical functions of the enzyme which are probably not targeted by conventional inhibitors.

In addition to its influence on leukemic stem cells, 5-LO is also involved in drug resistance in leukemic cells. The LT pathway mediates the BCR-ABL-independent resistance to Imatinib treatment in CML by downregulation of PTEN. Overexpression of the LT pathway was dependent on PKCβ. This study also showed that c-myb expression inversely correlates with PKCβ and 5-LO [77]. This is in accordance with studies demonstrating that expression of 5-LO is attenuated by c- and b-myb in monocytes/macrophages and colon cancer cells, respectively [78–80].

In contrast to its leukemogenesis-promoting role, 5-LO can also play an anti-tumorigenic role in leukemias. 5-LO was found to be down-regulated in leukemic blasts of MLL-rearranged AML being negatively correlated with Stat and k-Ras signaling. Here, 5-LO had anti-tumor and drug-sensitizing effects [81]. Furthermore, another report could show that at time of diagnosis levels of 5-LO are below normal in CML. Treatment with the tyrosine kinase inhibitor Imatinib restored 5-LO levels to normal in therapy responders. In patients destined to blast crisis, Imatinib treatment had no impact on 5-LO levels suggesting that the failure of restoring 5-LO expression may indicate a risk of disease progression [76].

### Solid tumors

#### 5-LO in tumor-associated leukocytes

**Fig. 3:**
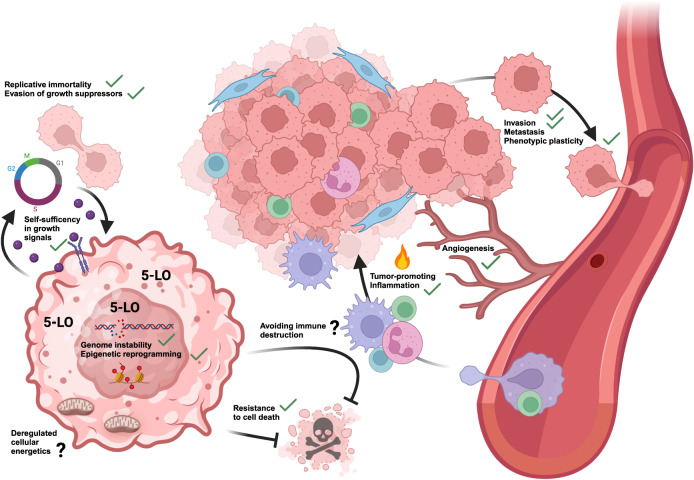
Influence of 5-LO on cancer hallmarks. The hallmarks influenced by 5-LO metabolites or the enzyme itself are marked with a green tick. The effects of 5-LO on cancer cell functions and the anti-tumor immune response appear to be an amalgam of canonical and non-canonical functions.

LTs and 5-oxo-ETE play an important role in innate immunity by inducing leukocyte migration and chemotaxis as well as microbial killing, thereby enhancing host defense. In addition, LTs activate pattern recognition receptors and the inflammasome [3, 82]. These pro-inflammatory properties suggest that the presence of 5-LO in the tumor tissue is unfavorable for solid tumors (Fig. 3). Indeed, a number of studies show that inhibition or knock-down of 5-LO and LT receptors exacerbates tumor growth in men and mice by attenuation of the anti-tumorigenic immune response. In a syngeneic mouse model of B16 melanoma, BLT1 KO mice show accelerated tumor growth and lower survival accompanied by a reduction in tumor-infiltrating CD8^+^ T cells. Adoptive transfer of wild type CD8^+^ T cells reduced tumor growth and augmented the response to immune checkpoint inhibitors [83]. Furthermore, mice that develop spontaneous colon cancer (APCmin/+ model) also show increased tumor development and mortality upon BLT1 deficiency. Interestingly, germ-free BLT1 deficient mice did not develop tumors at all and tumor development was dependent on MyD88 signaling suggesting an interplay of gut microbiota and LTs in colon tumor development [84]. Consistent with these findings, a clinical trial investigating the BLT1 inhibitor LY293111 for the treatment of lung cancer showed that inhibition of LTB_4_ signaling can exacerbate tumor progression [85]. These data clearly show that 5-LO products such as LTB_4_ play an important role in enhancing the patient’s tumor immune defense.

The inhibitor and knock-out data discussed above fit well to studies showing that tumors can actively silence 5-LO to evade immune destruction. In an immunocompetent mouse model of lung cancer, 5-LO expression is attenuated in tumor-associated macrophage populations [86]. In accordance, another study found out that co-culture of apoptotic cancer cells with macrophages reduces 5-LO expression in the latter in a c-myb-dependent manner, resulting in a reduced capacity to recruit T cells thereby promoting tumor progression [79].

In addition to the immune activating, anti-tumorigenic properties of 5-LO products derived from tumor invading leukocytes, 5-LO-derived oxylipins can also promote tumorigenesis (Fig. 3). LTs, 5-HETE and 5-oxo-ETE induce cancer cell proliferation and inhibit apoptosis thereby promoting tumor growth and cancer cell survival [87–95]. 5-oxo-ETE is also involved in cancer cell migration by triggering actin cytoskeletal changes and supports communication between cancer cells [96–98]. Furthermore, 5-LO products are involved in tumor angiogenesis and metastasis in vivo [99–101]. In line with this, neutrophil-derived LTs drive breast cancer metastasis by supporting the expansion of a sub-pool of highly tumorigenic cells [102].

#### Tumor cell-derived 5-LO

In addition to tumor-associated immune cells, sometimes the tumor cells themselves express high amounts of 5-LO. The enzyme has been detected among others in tumors of the human central nervous system, the gastrointestinal tract and the reproductive organs [103–108]. The tumor cell-specific 5-LO expression is quite remarkable, as the enzyme’s expression is usually restricted to leukocytes in healthy individuals. The non-physiological expression of 5-LO in the tumor itself is positively correlated with tumor growth and microvessel density as well as the metastatic potential of the malignant cells thus worsening the prognosis of the tumor as well as overall patient survival [108–112]. In addition, tumor cell-derived 5-LO expression has been associated with a poorer response to cytostatic therapy [113, 114].

Supporting these findings, many in vitro studies employing 5-LO-expressing cancer cell lines showed that 5-LO inhibitors trigger cell-cycle arrest and induce apoptosis in 5-LO-expressing cancer cells. Accordingly, add-back of 5-LO products rescues these effects [115–117]. Unfortunately, the interpretation of these pharmacological experiments is complicated: 5-LO inhibitors frequently used in literature such as Zileuton, AA-861 and others as well as inhibitors of LT receptors exhibit a wide range of pleiotropic effects, including inhibition of prostaglandin and EET formation and activation of PPARs [118–122]. The concentrations required to inhibit 5-LO activity / signaling are very close to those needed to elicit the pleiotropic effects, so off-target responses cannot be avoided. Indeed, it was shown that a number of 5-LO inhibitors attenuate cancer cell viability in a 5-LO-independent manner [123]. To further complicate matters, 5-LO-expressing tumor cell lines release only minute amounts of 5-LO products in vitro and hardly respond to established stimuli for LT formation such as Ca^2+^ ionophore and fMLP although the enzyme is active when partially purified [78, 124]. Therefore, add-back experiments are of little use to answer the question why these cells overexpress 5-LO.

In principle, it is not surprising that tumor cells silence 5-LO activity, as 5-LO products such as LTA_4_ and fatty acid hydroperoxides are known to form DNA adducts [125–127], signal ferroptosis [128–130] and may alert an anti-tumorigenic immune response. In line with this, it was recently shown that ER stress and chemotherapeutic agents induce LTC_4_ biosynthesis by upregulation of MGST2 together with 5-LO which then triggers ROS accumulation and oxidative DNA damage in WISH epithelial cells [131]. Furthermore, 5-LO activity can trigger cellular senescence by stabilizing p53 via a ROS-dependent phosphorylation in fibroblasts thereby reducing cell proliferation and regulating the cellular lifespan [132].

Interestingly, several studies could show that cancer cells upregulate 5-LO levels under cell stress conditions, while expression of the enzyme is suppressed when oxygen and nutrients are aplenty: Genotoxic stress triggered by treatment with cytostatic drugs activates the central tumor suppressor p53, a transcription factor that acts as a cellular stress sensor, tightly controls genomic stability and cell cycle progression and thereby regulates cell death and differentiation. Several reports show that p53 activation induces 5-LO expression. This is facilitated by binding of p53 to a transcriptional enhancer region in intron G of the 5-LO gene [133]. Furthermore, 5-LO co-localizes with p53 in cancer cells upon treatment with cytostatic drugs and alters the expression of p53 target genes suggesting a feedback loop between 5-LO and p53. In addition, it has been reported that upregulation of 5-LO upon genotoxic stress altered p53 nuclear trafficking in a 5-LO product-dependent manner leading to inhibition of apoptosis [134].

We could recently show that cell stress conditions that trigger G0/G1 cell cycle arrest in cancer cells such as three-dimensional growth as multicellular spheroids, low pH or very dense growth upregulates 5-LO expression in a p53-independent manner. The proliferating cancer cells suppressed 5-LO expression by a PI3K/mTORC2- and Mek-1/ERK-dependent axis involving b-myb. This upregulation of 5-LO was accompanied by a moderate increase in 5-LO activity [78]. An upregulation of 5-LO expression upon spheroid growth has also been reported for human glioma spheroids [135]. Furthermore, it was shown that pro-proliferative pathways suppress 5-LO in MLL-AF9 leukemic blast cells. Here, 5-LO expression was negatively correlated to Stat and k-Ras signaling [81].

It seems that 5-LO expression is higher under cell stress conditions in solid tumors (Fig. 3). But why do solid cancer cells upregulate a potentially harmful enzyme? The answer can only be: survival advantage. So, which survival advantage does 5-LO confer on the tumor cells and how?

Pharmacological studies cannot answer these questions due to the pleiotropic nature of LT pathway inhibitors already discussed above. A small number of studies employed alternative methods to attenuate 5-LO function in cancer cells: Using anti-sense technologies such as RNAi to knock-down 5-LO protein, it was shown that the enzyme influences tumor cell proliferation and viability [113, 117, 136–139]. Tumor cell functions such as migration, invasion and establishment of multicellular spheroids were not investigated in these studies. Having in mind the low overall activity of 5-LO in the tumor cells, we have recently established a CRISPR/Cas-mediated knock-out of 5-LO in two colon cancer and an osteosarcoma cell line and performed RNA-Seq to investigate if non-canonical activities of 5-LO might influence the overall gene expression in these cancer cells and thus cancer cell function. Our study showed that the influence of 5-LO on cancer cell gene expression and function seems to be an amalgam of canonical and non-canonical activities and is highly dependent on the cellular layout. We found that expression of 5-LO negatively influences tumor cell motility, fosters cell proliferation as well as invasion and multicellular spheroid formation by influencing genes of the cytoskeleton and extracellular matrix components suggesting an involvement in epithelial to mesenchymal transition and tumor mass maintenance. Furthermore, the expression of cytokines and growth factors such as MCP-1, TGFβ_2_, PDGFα and fractalkine as well as cell adhesion factors was influenced suggesting that 5-LO is also involved in the recruitment and manipulation of the tumor stroma [124]. Indeed, it has been recently reported that non-metastatic breast cancer cells express FLAP, LTC4S and 5-LO in contrast to their metastatic counterparts. Conditioned media of these cells induce regulatory B cells in a PPARα-dependent manner via 5-LO metabolites thereby reducing the number of T cells and facilitating murine breast cancer metastasis [140]. Another study has shown that hypoxic areas of human ovarian tumors express 5-LO and that this expression correlates with tumor progression and tumor-associated macrophage numbers [141]. Figure 3 comprehensively summarizes the influence of 5-LO on tumor cell function in solid tumors.

## Perspective

Although there is strong evidence that 5-LO plays an important role in the development of cancer, the global inhibition of LT formation and signaling has not yet shown the desired success in clinical trials. The inhibitors lacked clinical efficacy or even worsened patient outcome. This is probably due to the fact that global inhibition of LT formation affects not only tumor-derived 5-LO but also the leukocytic enzyme at the same time, thus impairing the anti-tumor immune response of the patients. Recent data show now that in addition to 5-LO-derived oxylipins, a number of non-canonical activities of 5-LO seem to have a major influence on cancer cell function and the microenvironment of tumors. Therefore, further studies should focus on pharmacological strategies that specifically target these non-canonical functions of 5-LO or specifically interfere with tumor cell-derived 5-LO expression while sparing the leukocytic enzyme. Furthermore, a deeper understanding of the 5-LO orchestrated tumor-immune interactions is needed to develop new therapeutic strategies for the treatment of 5-LO expressing solid tumors.
